# Difficult differential diagnosis of ectopic germinoma from multiple sclerosis: A case report and literature review

**DOI:** 10.1016/j.ijscr.2023.107884

**Published:** 2023-01-13

**Authors:** Keisuke Masuda, Nayuta Higa, Hajime Yonezawa, Hiroyuki Uchida, Ryosuke Hanaya

**Affiliations:** Department of Neurosurgery, Graduate School of Medical and Dental Sciences, Kagoshima University, 8-35-1 Sakuragaoka, Kagoshima 890-8520, Japan

**Keywords:** ASL, arterial spin labeling, CNS, central nervous system, CSF, cerebrospinal fluid, OCBs, immunoglobulin oligoclonal bands, MS, multiple sclerosis, MR, magnetic resonance, SWI, susceptibility-weighted imaging, Primary frontal germinoma, Ectopic germinoma, Central dilated vascular structure, Multiple sclerosis, Case report

## Abstract

**Introduction and importance:**

Intracranial germinomas are germ cell tumors that commonly develop in the pineal or neurohypophysis regions. As ectopic germinomas are rarely observed within the cerebrum and are associated with atypical image findings, diagnosis is challenging.

**Case presentation:**

A 14-year-old boy was admitted to our hospital with complaints of vomiting and headache. Gadolinium-enhanced magnetic resonance imaging revealed ring-enhancing lesions in his left frontal lobe and basal ganglia. Susceptibility-weighted imaging indicated that the subependymal veins passing through the lesion centers were engorged, while electrophoretic analysis of cerebrospinal fluid identified oligoclonal bands (OCBs); both were typical of multiple sclerosis (MS). Tumor biopsy revealed many cells with atypical mitotic figures and nuclear enlargements, suggesting malignant disease. As the tumor rapidly proliferated, we opted for surgical excision of the lesions. Histopathological analyses revealed “two-cell patterns” characteristic of germinoma. Immunohistochemistry was positive for placental alkaline phosphatase and c-KIT. The definitive diagnosis was germinoma. After chemoradiotherapy, the patient was discharged without neurological deficits.

**Clinical discussion:**

OCBs and several magnetic resonance imaging features (including open ring enhancement, T2 hypointense rims, mild mass effects, mild perilesional edema, peripheral restriction around the lesion, and vessel-like structures running through the lesion center) are useful diagnostic signs for the radiological discrimination of MS from germinoma. However, owing to these factors, some cases are difficult to diagnose.

**Conclusion:**

Our case report of an unusual ectopic cerebral germinoma illustrates the difficulty of distinguishing it from MS. Therefore, we recommend proper tissue sampling in such cases, especially in adolescent patients, to make definitive germinoma diagnoses.

## Highlights

• We report a rare case of intracranial germinoma in the frontal lobe and basal ganglia.
• The engorged subependymal vein in the lesion center is atypical in intracranial germinoma.
• Proper tissue sampling is required for germinoma diagnoses, especially in adolescents.

## 1 Introduction

Intracranial germinoma is a type of germ cell tumor occurring in the central nervous system (CNS). These tumors predominantly develop during adolescence and account for approximately 10 % of pediatric brain tumors in Japan [1], but only 4 % in Europe and the Americas [2]. Over 80 % of germinomas originate within either the midline structures, pineal gland, or neurohypophysis [3]. Approximately 10 % of intracranial germinomas occur ectopically; such germinomas tend to arise within the basal ganglia or thalamus and have worse outcomes [1]. Periventricular white matter ectopic germinomas are much rarer, but the cause remains unknown.

Tumefactive multiple sclerosis (MS) is a disease characterized by tumor-like lesions in the CNS that are generated by the demyelinating process. The presence of immunoglobulin oligoclonal bands (OCBs) isolated from cerebrospinal fluid (CSF) and several radiological observations are useful for differentiating MS from germinomas [4], [5]. In this report, we describe the case of a 14-year-old boy with ectopic germinomas localized in the white matter of the frontal lobe that mimicked tumefactive MS and review similar cases in the literature. The study has been reported in line with the SCARE criteria [6].

## 2 Presentation of case

**Fig. 1:**
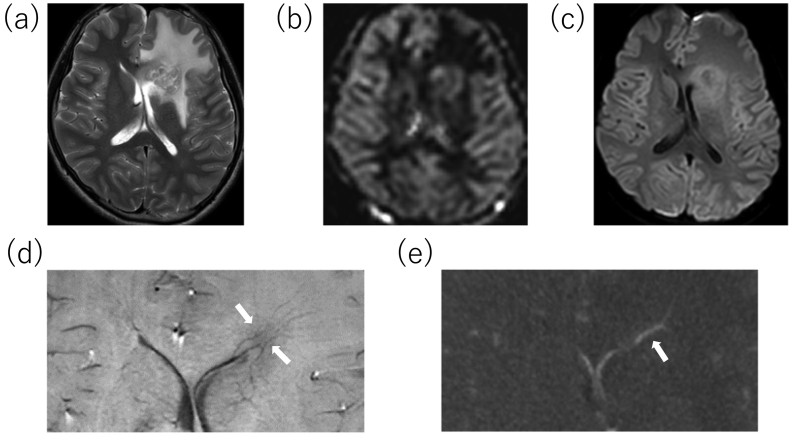
(a) On T2-weighted images, lesions appear hypointense and surrounded by extensive edema with no hypointense rim. (b) Arterial spin labeling image shows increased blood flow only in the merged area, not in the internal lesion. (c) Peripheral restriction around the lesion shown on a diffusion-weighted image. (d) Susceptibility-weighted image shows that veins passing through the lesion centers are visibly engorged (arrows). (e) Contrast-enhanced computed tomography image also reveals the engorged central vein (arrow).

**Fig. 2:**
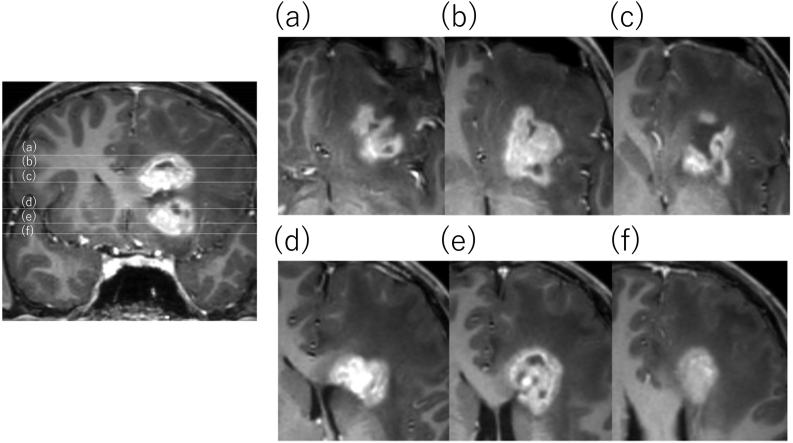
Gadolinium-enhanced magnetic resonance images. Two ring-enhancing lesions can be seen forming a unique shape that straddles the anterior horn of the left lateral ventricle: the inferior lesion extends from the anterior corpus callosum to the deep white matter, and the superior lesion extends from the basal ganglia to the orbital gyrus (each 3 cm in maximum diameter). A coronal section (left) and an axial section for each line drawn in the coronal section (right, a–f) are shown.

**Fig. 3:**
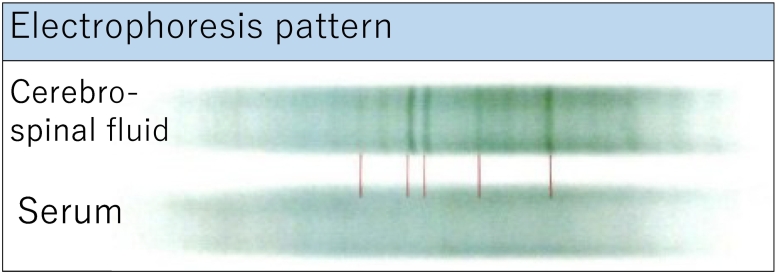
By immunofixation electrophoresis, five oligoclonal bands were detected in cerebrospinal fluid.

A 14-year-old boy with headaches and nausea was admitted to our hospital following referral by his family doctor. On admission, the patient was lucid with no obvious neurological deficits. The patient had no relevant medical history, family history, or drug history. Magnetic resonance (MR) T2-weighted imaging revealed extensive edematous changes to his left frontal lobe (Fig. 1a) with no hypointense rim. The blood levels of alpha-fetoprotein and human chorionic gonadotropin β-subunit were within normal limits. Gadolinium contrast-enhanced MR imaging revealed two irregular ring-enhancing lesions around the anterior horn of the left lateral ventricle (Fig. 2). The superior lesion was including the globus pallidus and the inferior lesion was on the white matter of the frontal lobe. There was no lesion within the midline structures. Arterial spin labeling (ASL) and MR perfusion showed increased blood flow only in the merged area, not in the internal lesion (Fig. 1b). Peripheral restriction around the lesion was shown on diffusion-weighted images (Fig. 1c). Susceptibility-weighted imaging (SWI) indicated that the subependymal veins passing through the centers of the lesions were engorged (Fig. 1d). The engorged veins were also visible on Contrast-enhanced computed tomography imaging (Fig. 1e) Based on these atypical findings, the neuroradiologist listed tumefactive MS as the leading differential diagnosis. Although we were aware of the risk of tonsillar herniation, as it was deemed essential to perform a cerebrospinal fluid examination to diagnose MS, we carefully performed a lumbar puncture but drained only 3 mL of CSF to avoid the risk of cerebral herniation. The pressure of CSF was 255 mmH_2_O. The CSF samples showed an elevated protein level (105 mg/dL) and an unremarkable cell concentration and immunoglobulin G index (1.01). Cytological diagnosis could not be performed because it was not possible to drain the required amount of CSF. We observed five unique OCBs, which indicated MS (Fig. 3).

**Fig. 4:**
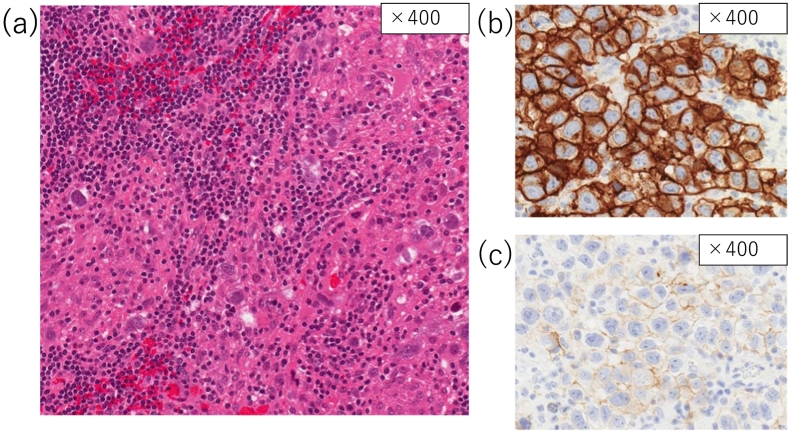
(a) Hematoxylin and eosin-stained section (×400 magnification). The characteristic “two-cell pattern” histopathology of germinoma is apparent from the different-sized cells. Immunohistochemistry (×400 magnification) was positive for c-KIT (b) and placental alkaline phosphatase (c).

These findings were consistent with tumefactive MS; however, glioblastoma or other neoplastic tumor remained a differential diagnosis. Therefore, we performed a stereotactic tissue biopsy to confirm the histopathological diagnosis. Even after the biopsy, we could not attribute a definitive diagnosis; nevertheless, the presence of atypical mitotic figures in the biopsy specimen strongly suggested malignancy. As the patient's condition worsened over the subsequent month, we opted to surgically excise the lesions. Because intraoperative rapid diagnosis was not sufficient to determine the diagnosis, we opted for subtotal resection of the tumor. Histopathological analyses of the excised tumor cells using hematoxylin and eosin stain revealed a “two-cell” cytological pattern, along with large round nuclei and pale and foamy cytoplasm against a backdrop of extensive lymphocyte infiltration (Fig. 4a). The tumor was immunoreactive for c-KIT (Fig. 4b) and placental-like alkaline phosphatase antibodies (Fig. 4c), with a very high ratio of Ki-67-positive cells (80 %). Histopathology confirmed the diagnosis of germinoma.

We subsequently administered postoperative chemoradiotherapy, a combination of three cycles of carboplatin/etoposide therapy (carboplatin 600 mg/m^2^/day+etoposide 100 mg/m^2^/day), and whole-brain irradiation (23.4 Gy in 13 fractions). Over the 6-month follow-up, the patient remained recurrence-free, performing activities of daily living without neurological deficits.

## 3 Discussion

To date, only 13 cases of primary frontal germinoma have been reported in the literature, (Table 1) [7], [8], [9], [10], all of which are from Asian countries. Among the reported cases, 80 % of patients underwent total or subtotal resections, although intracranial germinomas do not require resection. Such findings indicate that preoperative diagnosis of frontal germinoma is challenging.

**Table 1:** List of reported cases of primary frontal lobe germinoma.

	Age/sex	Symptoms	Tumor location	Enhancement	Therapy	Prognosis (time)
Luo et al. [8]	10/M	Hemiplegia	Left frontal	NA	Ope (STR), radiation	Dead 7 months
	15/M	Somnolence, hemiplegia	Left frontal	NA	Ope (STR), radiation	Dead 9 months
	7/M	Hemiplegia	Right frontal	NA	Ope (STR), radiation	Alive 16 years
Luo et al. [8]	20/M	Headache, vision loss	Bilateral frontal	NA	Ope (STR), radiation	Alive 3 years
Luo et al. [8]	58/F	Hemiplegia	Bilateral frontal	NA	Ope, radiation	Alive 9 months
Luo et al. [8]	12/M	Hemiplegia	Right radial crown	Heterogeneous enhancement	Ope (STR), chemo, radiation	Dead 14 months
Luo et al. [8]	12/F	Hemiplegia	Left frontal, basal ganglia	No enhancement	Ope (STR), radiation	Alive 4 years
Luo et al. [8]	20/M	Headache, apnea	Right frontal	NA	NA	Dead 5 days
Utsuki et al. [7]	22/M	Memory impairment, abnormal behavior	Corpus callosum, right frontal	Heterogeneous enhancement	Ope (STR), radiation, chemo	Alive 2 years
Luo et al. [8]	19/M	Headache, nausea	Left frontoparietal	Slight enhancement	Ope (TR), radiation	Alive 3 years
Luo et al. [8]	19/M	Headache, nausea	Left frontal	Heterogeneous enhancement	Ope (TR), radiation	Alive 3 years
Wang et al. [9]	21/M	Seizure	Frontal	No enhancement	Ope (STR), radiation, chemo	Alive 6 months
Caro-Osorio et al. [10]	17/F	Personality change, loss of appetite	Corpus callosum, bilateral frontal	NA	Biopsy, radiation	Alive 2 years
Present case	14/M	Headache, nausea	Left frontal, basal ganglia	Ring enhancement	Ope (STR), radiation, chemo	Alive 6 months

M, male; F, female; NA, not available; Ope, operation; TR, total resection; STR, subtotal resection; Chemo, chemotherapy.

OCBs are bands of immunoproteins generated in the CSF and are clinical hallmarks of MS. However, OCBs have also been reported in patients with germinoma [11]. Several conventional MR imaging features (including open ring enhancement, T2 hypointense rims, mild mass effects, mild perilesional edema, and peripheral restriction around the lesion on diffusion-weighted images) are useful diagnostic signs for the radiological differentiation of MS from neoplastic lesions [12]. In the present case, T2 hypointense rims were not detected. Although strong perilesional edema and mass effects of the lesion suggested malignant disease, peripheral restriction around the lesion indicated MS.

ASL and MR perfusion reveal increased blood flow in neoplastic diseases but not in demyelinating diseases [12], [13]. In our case, ASL and MR perfusion showed increased blood flow only in the merged area, not in the internal lesion, suggesting malignancy.

The presence of engorged veins running through the lesion center, as visualized via SWI, can be useful for differentiating MS from neoplastic lesions [4], [12], [14]. To date, no reports on lesional venous engorgement in germinoma have been published, contributing to the difficulty in our initial diagnosis. Importantly, the present case shows that lesional venous engorgement can also occur in germinoma.

Several case reports have recommended biopsies or therapeutic diagnosis of high doses of steroids as the next step for diagnosis [4], [5], [15]. We selected a biopsy for our patient, as the use of steroids causes a delay in the diagnosis of some types of tumors. The stereotactic needle biopsy was a useful indicator of malignancy but was insufficient to diagnose germinoma definitively. As these conventional methods such as stereotactic needle biopsy or open biopsy have some advantages and disadvantages in sampling accuracy, approach to deep lesions, sample volume, and invasiveness. The choice of optimal technique depends on tumor location and preoperative diffusion tensor image examination [4], [16]. In this case, the lesion was in the left frontal lobe and there is a low possibility of neurological dysfunction due to tract for reaching the lesion. In addition, because of this unusual imaging findings and examination findings, there is a possibility that a sufficient diagnosis cannot be made with biopsy. Considering these, it should be considered as one means to perform an open biopsy.

## 4 Conclusion

In conclusion, although ectopic germinomas are rare, they should be included in the differential diagnosis of younger patients with imaging findings suggestive of demyelinating disease to ensure proper treatment strategies are administered before a biopsy is performed.

## Consent

Written informed consent was obtained from the patient's parents for publication of this case report and accompanying images. A copy of the written consent is available for review by the Editor-in-Chief of this journal on request.

## Ethical approval

Ethical Approval was waived by the authors institution.

## Funding

None.

## Guarantor

Nayuta Higa, M.D., Ph.D.

## Research registration number

1. Name of the registry: None
2. Unique identifying number or registration ID: None
3. Hyperlink to your specific registration (must be publicly accessible and will be checked): None.

## CRediT authorship contribution statement

Keisuke Masuda, Nayuta Higa substantially contributed to the manuscript drafting. Keisuke Masuda, Nayuta Higa and Hiroyuki Uchida treated and operated on patients. Hajime Yonezawa and Ryosuke Hanaya analyzed and interpreted the patient data. All authors read and approved the final manuscript.

## Declaration of competing interest

None.

## Data Availability

Not applicable.
